# A Bill to Regulate the Practice of Dentistry

**Published:** 1866-07

**Authors:** 


### Editorial.

### A BILL TO REGULATE THE PRACTICE OF DENTISTRY.

At the last session of the Legislature of Ohio, a considerable number of petitions were presented to that body from members of the profession and the people at large, praying for such a law, or laws, as would, so far as possible, protect the people from dental quackery—from those who are manifestly deficient in ability, and influenced only by a desire for gain. Hundreds and thousands are made to suffer incalculably, by the practice of incompetent and mercenary men, who, without any proper knowledge, attempt to discharge the important, responsible, and difficult duties devolving upon the dental surgeon.

The object of such a law is two-fold. In the first place to protect the people from the worse than worthless operations of incompetent and unprincipled dentists; and second, to prevent, so far as possible, the incompetent from entering the practice of the profession. Such an enactment could in no sense operate oppressively upon the competent, but would certainly be a great stimulant to all such. The simple legal recognition of ones ability would be, to say the least, gratifying and encouraging.

Could it be possible that the ranks of the profession were to be occupied only by those of professional efficiency, the emulation that would thus be aroused can scarcely be imagined; then, indeed, would there be progress. Quackery serves almost as a dead lock upon the profession. It is perhaps true that one here and another there rises, to some extent, above its blighting influence, and by effort, involving a constant struggle, accomplishes something in the way of progress; yet, none are wholly free from its influence, and the greater proportion of the worthy part of the profession, are so hedged about by this incubus, as to be unable to move in the direction of progress. No one who is competent and successful in his practice, can, with reason, do less than sustain and labor for such a law; and no one with intelligence and good understanding among the people, will fail, for a moment, to sustain and advocate such a measure, when they clearly understand it.

No one can, with any propriety, oppose such a law, except the incompetent and quack. If by the legal knife, even a portion of the foul excrescence, that hangs like a body of death upon our noble profession, can be cut off, let it be exercised. But it is said that by this means but a part at best can be separated; that may be true, so it is also true that a man may swim with a twenty pound weight attached to him, while with fifty pounds he would sink and be drowned, indeed one pound may make the difference of sink or swim.

It has been suggested that such a law would not be enforced. We, wot of a few, who would be exceedingly glad of an opportunity to enforce such a law just now, a few who have become conscious that they have been made miserable for life, by mal-practice. There will be no more difficulty in its enforcement, than in that of any wholesome regulation. It is a law that none but the evil doers will have occasion to fear. It would, emphatically, be a terror to evil doers, and a praise to them who do well.

But it is said that "the people have a perfect right to select their physician or dentist." So they have, so long as that selection is made from those who are competent; but they have no more right to select one who would do them injury, or ruin their health, than they have to take arsenic, or strychnia, or shoot themselves; and the only apology that is possible to make for any one making choice of an incompetent dentist, is that they are ignorant.

But the principle, that ignorance should not tamper with dangerous things, is fully recognized in the legal restrictions that are placed upon the sale of poisons, gunpowder, &c. It is said that "the presumption is that any one who assumes a responsible position fits himself for that position." Yes! and that is about as true as that every man we meet is just what he ought to be. Everybody knows, that in all places there are men who are *not* what they assume to be, and not what others may *presume* them to be.

We propose, in the next number, to pursue this subject further, and consider what quackery has done and is doing, and the advantages that would accrue from a proper restrictive law.

We give, below, the Dental Bill, proposed, presented, and considered, in the Senate of the Ohio Legislature, at its last meeting. And we ask for it a careful consideration by the profession, and if in any respects it can be improved, let it be done—at least if it is thought advisable to again ask our Legislature for its passage. Other States are moving in the same matter, and if there is anything desirable in it, it will be well that our State should first reap the benefit; having first put the ball in motion.

### "A BILL—TO REGULATE THE PRACTICE OF DENTISTRY IN THE STATE OF OHIO."

SECTION 1. *Be it enacted by the General Assembly of the State of Ohio,* That after the first day of September, 1866, it shall be unlawful for any person to practice dentistry in the State of Ohio, unless such person has received a diploma from the faculty of a dental college duly incorporated under the laws of this or any other State of the United States, or a certificate of qualification from the board of examiners, or any citizen of this State who has been in reputable practice of dentistry for the term of ten years.

SEC. 2. Said board of examiners shall be appointed by the governor, by and with the consent of the senate, and consist of three practitioners of dentistry, who shall be regular graduates of a dental college duly incorporated under the laws of this or any other State of the United States, or persons possessing the evidence of qualification contemplated in this act.

SEC. 3 The board of examiners shall serve for a term of three years, and until their successors are qualified, excepting the members of the first board, one of whom shall serve for one year, one for two years, and one for three years.

SEC. 4. The board of examiners shall meet at the capital of the State at least once a year for the purpose of examining applicants, after having given at least sixty days notice of such meeting in some newspaper of general circulation throughout the State. They shall also have power to make such arrangements as shall be necessary for the prompt and efficient performance of their work as such examiners.

SCE. 5. Each applicant shall, on receiving a certificate, pay into the treasury of the board, the sum of ten dollars, which shall constitute a fund to defray the expenses of the board, and out of the fund thus created, each member of said board of examiners shall receive the sum of ten dollars per day, for services rendered as such examiners.

SEC. 6. Any person who shall practice dentistry without having complied with the requisitions of this act, shall be deemed guilty of a misdemeanor, and upon conviction thereof, shall be fined not less than fifty dollars, nor more than two hundred; provided, that nothing in this act shall be construed to prevent physicians and surgeons from extracting teeth.

SEC. 7. All prosecutions under this act shall be by indictment before the court of common pleas in the county where the offence shall have been committed, and all fines imposed and collected under this act, shall be paid into the treasury of the county where such conviction shall take place, for the use of the common schools within such county.

SEC. 8. This act shall take effect and be in force from and after its passage.

